# Factors influencing pharmacokinetics of 5-fluorouracil in cancer patients: a systematic review of population pharmacokinetic models

**DOI:** 10.1007/s00228-026-04043-5

**Published:** 2026-04-15

**Authors:** Teshini Suthahar, Jayashree Veerabhadrappa, Renuka Munshi, Elstin Anbu Raj, Vikram Gota, Vijay Ivaturi, Surulivelrajan Mallayasamy

**Affiliations:** 1 Department of Pharmacy Practice, Manipal College of Pharmaceutical Sciences, Manipal Academy of Higher Education, Manipal, 576 104 Karnataka India; 2 Department of Clinical Pharmacology, TN Medical College & BYL Nair Hospital, Dr. AL Nair Road, Mumbai Central, Mumbai, 400 008 India; 3 Department of Health Technology and Informatics, Prasanna School of Public Health , Manipal Academy of Higher Education, Manipal, 576 104 Karnataka India; 4 Department of Clinical Pharmacology, Advanced Centre for Treatment, Research and Education in Cancer, Mumbai, India; 5 Centre for Pharmacometrics, Department of Pharmacy Practice, Manipal College of Pharmaceutical Sciences, Manipal Academy of Higher Education, Manipal, 576 104 Karnataka India

**Keywords:** Population Pharmacokinetics, 5-Fluorouracil, Non-linear mixed effects model, Pharmacokinetics, Systematic review

## Abstract

Purpose 5-Fluorouracil (5-FU) is a traditional chemotherapeutic agent used in the treatment of multiple cancers. This systematic review aimed to summarize the population pharmacokinetic (PopPK) models for both intravenous and pro-drug formulations in cancer patients and to understand the different covariates that affect the pharmacokinetics of 5-FU. Methods the search included studies published from inception to December 2025 in the English language. The non-linear mixed effects model with parametric approach for 5-FU in cancer patients was set as the inclusion criteria. A total of 186 articles were screened for their titles and abstracts from Scopus, PubMed and EMBASE. Out of which 156 were excluded. Out of the 36 articles reviewed, 21 were found suitable for the review. Most of the studies reported a one-compartmental model. Several covariates, including body surface area (BSA), body weight, cancer type, age, gender, UH2/U ratio, serum albumin, creatinine clearance, alkaline phosphatase, total bilirubin, and skeletal muscle index (SMI), influence the pharmacokinetic parameters, including clearance and volume of distribution. Results skeletal muscle index (SMI), body weight, sex, and BSA were the primary covariates influencing the pharmacokinetics of 5-FU. Among the covariates consistently reported as significant, sex was an important determinant of clearance, with males exhibiting higher clearance than females. Increases in body weight and skeletal muscle index were associated with increased clearance, while volume of distribution decreased with lower body surface area. Alkaline phosphatase was reported to have a negative effect on clearance. The substantial interindividual variability observed across studies likely contributes to heterogeneity in reported covariate effects and underscores the need for individualized dosing approaches for 5-FU. Conclusion body weight and sex reported to show clinical significance. This necessitates the need for consideration of these covariates in dosing adjustment. This review summarizes covariates influencing the pharmacokinetics of 5-FU, and reports those identified as significant based on forest plot analysis. This study helps the researchers to perform predictive performance of the reported PopPK models, supporting future model-informed precision dosing strategies for 5-FU-based therapies.

**Supplementary Information:**

The online version contains supplementary material available at 10.1007/s00228-026-04043-5.

## Introduction 

5-Fluorouracil (5-FU) persists as a fundamental chemotherapeutic agent for over six decades following its clinical introduction. Despite the advent of novel targeted therapies and immunotherapies, 5-FU remains extensively utilized, either as a solitary treatment or in combinatorial regimens such as FOLFOX, FOLFIRI, and CAPOX for the management of colorectal, breast, gastric, pancreatic, and head and neck malignancies. Its enduring clinical significance is attributable to its economic viability, well-defined pharmacological mechanism, and established effectiveness across a diverse array of tumour types [1, 2].

5-FU is an analogue of pyrimidine uracil used in the treatment of solid tumours and exerts its cytotoxic effect by inhibiting thymidylate synthase, an enzyme that contributes to DNA replication [3]. It is administered as intravenous (*i.v)* bolus(500 mg/m^2^), continuous infusion and orally as pro-drugs including capecitabine, tegafur/gimeracil/oteracil (S-1), and tegafur/uracil (UFT) [4].It is a narrow therapeutic index drug with a target of AUC approximately 20–30 mg. h/L and high interindividual variability (IIV) ranging in pharmacokinetics (PK) and toxicity profiles, which limits its therapeutic use [5]. The enzyme dihydropyrimidine dehydrogenase (DPD), which is encoded by the dihydropyrimidine dehydrogenase (DPYD) is responsible for 80% metabolism of 5-FU. Variants in DPYD are associated with reduced or absent enzyme activity, leading to impaired 5 fluorouracil clearance and increased risk of toxicity. The partial or complete deficiency is identified to be contributing to increase in 5-FU plasma concentrations and associated toxicities such as mucositis, myelosuppression, and gastrointestinal disturbances [6]. Over 35 genetic variations in DPYD have been identified, making it a highly polymorphic gene. Several of these variations result in decreased function of DPD enzymes including DPYD*2A, c.2846 A > T, c.1679T > G, and c.1236G > A [7].

Due to variations in their activation mechanisms and DPD activity regulation, oral 5-FU pro-drugs such as capecitabine, S-1, and UFT have different dose regimens. When delivered as part of the oral combination agent S-1, which includes the DPD inhibitor, 5-chloro-2,4-dihydroxypyridine(CDHP) to modulate systemic exposure, the typical 5-FU equivalent dose ranges from 25 to 45 mg/m²/day [8]. In contrast, capecitabine, another oral 5-FU pro-drug that undergoes a three-step enzymatic conversion to 5-FU without co-administration of a DPD inhibitor. Therefore, capecitabine is typically administered at a substantially higher dose of approximately 1250 mg/m² twice daily for 14 days, followed by a 7-day break, making it more vulnerable to fluctuations in DPD function [9]. To decrease 5-FU breakdown, however, DPD inhibitors such as uracil in UFT and CDHP in S-1 are co-administered. This results in less toxicity and more predictable exposure in individuals with normal DPD activity [10, 11].The standard dosing is based on Body Surface Area (BSA, mg/m^2^) strategy. However, some studies reported no association between 5-FU pharmacokinetics and BSA supporting the need for Therapeutic drug monitoring (TDM) [12].

Non-genetic factors such as age, sex, renal and hepatic function, and nutritional status (e.g., skeletal muscle index (SMI)) have also impacted in 5-FU PK variability. The oral administration of 5-FU results in varying plasma levels due to variability in bioavailability and the rapid *i.v* bolus injection resulting in saturable metabolic process leading to disproportionate increases in 5-FU concentration. Infusions administered at usual doses of 300–500 mg/m2/day for 5 days show dose dependent linear disposition kinetics making it widely used in practice. The total clearance of 5-FU administered as continuous venous infusion (CVI) ranges from 120 to 360 L/hr with a half-life of 10–15 min with linear pharmacokinetics [13].

It has been reported that the patients under 5-FU therapy have higher (over 100%) difference in systemic exposure with lower therapeutic index [14]. This high inter individual variability in the pharmacokinetic parameters of 5-FU necessitates the need for Population pharmacokinetics (PopPK) models. These models aim to quantify the variability caused by different covariates in drug exposure observed among individuals within a given population. This approach estimates the population value (mean) of PK parameters, the IIV and the residual unexplained variability [15].

Despite the clinical evidence of covariates that affect the PK of the 5-FU from individual PopPK models, there remains no consolidated understanding of which patient-related covariates consistently and meaningfully explain variability in 5-FU exposure. The aim and objective of this systematic review was to identify and summarize the covariates that significantly affects the PK of 5-FU and to study how does that explain the variability. The information published on 5-FU PopPK models, including demographic characteristics, model parameters, covariate significance and their mathematical relations is reported and discussed.

## Methodology

### Search strategy

The systematic review was conducted in accordance with The Preferred Reporting Items for Systematic Reviews and Meta analyses (PRISMA). A systematic search was conducted in PubMed, Scopus and EMBASE databases. This systematic review included articles from inception until December 2025. The search keywords employed in the databases includes “Neoplasm” OR “Cancer” AND “fluorouracil” OR “5-fluorouracil” AND “population pharmacokinetic” OR “pop pk” AND “NONMEM”. Table 1 in online resource depicts the search strategy.

**Table 1:** Characteristics of 5-FU population pharmacokinetics studies

No	Reference	Model	Software; parameter estimation method	Method validation	Tested covariates	Addition/deletion of covariates	Parameter (significant covariates)
5-FU IV							
1	Marie Sandstrom et al., (1996)	One-compartment (non-linear elimination)	NONMEM, version IV; FO	Goodness-of-fit plots	No tested covariates	NR	NR
2	M.-C. Etienne et al., (1998)	One-compartment (first order elimination)	NONMEM program, version IV, level 1.1	Linear regression	Age, sex, BSA, hepatic metastases, PMNC-DPD, liver enzymes, clock-time (8 a.m. versus 5 p.m.), elapsed time during CVI	*P* < 0.05; *P* < 0.001	Cl (age, low PMNC-DPD, high serum ALP and elapsed time during infusion)
3	FrancË oise Bressolle et al., (1999)	One-compartment	NONMEM computer program (Version 4.0, level 2.1); FO	Bayesian	Sex, BSA, age, BW, H, liver enzymes and serum creatinine	*P* < 0.05; *P* < 0.05	CL(Sex)
4	M. climente-marti et al., (2002)	One compartment (first order elimination)	NONMEM program (version V, level 1.0); FOCE	Bayesian	Age, gender, BW, IBW, H, BSA, CL_CR_, and hepatic function tests	*P* < 0.01; *P* < 0.01	Vd(IBW) and CL(BW)
5	M.A. Batey et al., (2002)	One compartment	NONMEM software (version V); FOCE	NR	Age, BW, creatinine, TB, AST, ALT, ALP	NR	CL(Age)
6	M. Sandstrom et al., (2006)	One- compartment model with saturable elimination	NONMEM version VI; FOCE-I	Goodness-of-fit plots	Age, BW, H, BSA, AST, ALT, ALB, TB, creatinine, CL_CR_	*P* < 0.05; *P* < 0.01	V(BSA); Vmax(CL_CR_)
7	Nagulu Malothu et al., (2010)	One-compartment (first order elimination)	NONMEM (version 5); FOCE-I	Goodness-of-fit plots	BW, H, age, dose and gender	NR	No influence of covariates
8	F. Mueller et al., (2013)	One-compartment (first order elimination)	NONMEM version VII; FOCE-I	Goodness-of-fit plots and Internal bootstrap test	Gender, age, BSA, CLCR, AST, ALT, albumin, TB and the DPYD genotype	*P* < 0.01; *P* < 0.01	CL(Gender)
9	Eduard Schmulenson et al., (2022)	One-compartment model with linear elimination	NONMEM^®^ version 7.2; FOCE-I	Goodness-of-fit plots, Internal bootstrap test and prediction- corrected visual predictive check	Age, sex, infusion time, CR, TB, ALT, AST, GGT, LDH, tumor markers (CA 19–9, CEA), and BSA	*P* < 0.05; *P* < 0.01	CL(BSA, Skeletal muscle index)
10	B.Porta-Oltra et al., (2004)	Two-compartment (first order elimination)	NONMEM package version V level 1.0; ELS	Goodness-of-fit plots and Internal bootstrap test	Age, BW, gender, IBW, LBM, BSA, Quetelet index, ALT, AST, GGT, ALP, LDH, TB, DB, ALT/ALP quotient, CR	*P* = 0.01; *P* = 0.005	No influence of covariates
11	Christian Woloch et al., (2012)	Two-compartment model	NONMEM version VI; FOCE-I	Visual Predictive Check	Age, sex, BW, H, CL_CR_ AST, ALT, GGT, histological status, BSA, LBM	*P* < 0.001	No influence of covariates
12	Usman Arshad et al., (2020)	Two-compartment with linear elimination	NONMEM 7.4.2; FOCE-I	Visual predictive checks	Age, BW, H, sex, BMI, LBW, BSA, ALT, AST and GGT, and albumin; and DPYD, TS and MTHFR genotypes.	*P* < 0.05; *P* < 0.05	CL(BSA)
13	Akihiro Yamada et al., (2025)	One -compartment model with zero-order input and first-order elimination	NONMEM^®^ (version 7.5.0)	Visual predictive checks	Zolbetuximab effect	NR; *P* = 0.001	No influence of covariates
**5-FU PRO-DRUGS**							
14	R. Gieschke et al., (2002)	One- compartment model (nonlinear elimination)	NONMEM (Version 5, level 1); FO	Goodness-of-fit plots	Age, BW, gender, race, BSA, CL_CR_, Cancer type, Liver metastasis, TB	*P* < 0.05; *P* < 0.001	CL(Cancer type)
15	Ronald Gieschke et al., (2003)	One-compartment	NONMEM (Version 5, level 1); FO	Goodness-of-fit plots	Age, BW, BSA, CL_CR_, gender, Race (Caucasian/black/other), Karnofsky scale (70/80/90–95/100), Liver metastasis, Albumin, AST, ALT, ALP, TB	*P* < 0.05	CL(ALP)
16	Emmanuelle Comets et al., (2003)	One-compartment	NONMEM version 5.1; FOCE-I	Goodness-of-fit plots and pseudo-residuals	BSA, sex, age, cancer type	*P* < 0.01; *P* < 0.01	Vd (BSA)
17	Saik Urien et al., (2005)	One-compartment	NONMEM (version V, level 1.1); FO	Goodness-of-fit plots and Internal bootstrap test	BW, BSA, H, age, TB, serum albumin	NR	No influence of covariates
18	Hyeong-Seok Lim et al., (2015)	One-compartment	NONMEM program (version IV, level 1.1); FOCE-I	Goodness of fit and Visual Predictive check	Plasma CDHP concentrations, sex, age, BW, CL_CR_ based on serum CR, and CL_CR_ based on 24-hour urine CR	*P* < 0.05	No influence of covariates
19	Esther Oyaga-Iriarte et al., (2019)	One-compartment	NONMEM	Goodness-of-fit plots and Visual Predictive Check	NR	NR	NR
20	Nastja Lunar et al., (2021)	One-compartment	Monolix^®^ (version 2019R1); SAEM	Visual Predictive Check	DPYD genotype, age, U, UH2/U ratio, CDA activity in plasma, BSA, ALP, GGT, AST, ALT, sodium concentration, kaliemia, CR, TB, albumin, plasma proteins and time between breakfast and capecitabine	*P* < 0.01; *P* < 0.001	K_el_ (UH2/U ratio)
21	S.Urien et al., (2003)	Two-compartmental model	Micropharm Population, MP2	Goodness-of-fit plots	Age, BW, BSA, ASAT, ALAT, ALP and TB.	*P* < 0.01; *P* < 0.01	No influence of covariates

*GC-NICI-MS* Gas chromatography with negative ion chemical ionization mass spectrometric detection, *HPLC* High-performance liquid chromatography, HPLC with *UV* High-performance liquid chromatography with ultraviolet absorbance detection, *LC-MS/MS* Liquid chromatography–tandem mass spectrometry, *NR* Not reported, RP-HPLC with *UV* Reverse-phase HPLC with UV detection, ^#^- Mean

## Inclusion and Exclusion Criteria

A systematic search was undertaken independently by two reviewers (T.S and J.V) to screen the relevant literature. The screening on title, abstracts and full text was done by the reviewers on meeting the eligibility criteria, (1) The patients under 5-FU therapy; (2) PopPK models using non-linear mixed effect modelling approach; (3) Published in English; (4) Studies conducted in humans.

The exclusion criteria include, (1) Review papers, case reports (2) Unretrievable publication (3) Unavailability of final model parameters of 5-FU.

## Identification of Studies

The search results were exported to Rayyan software [16]. The de-duplication process was carried out using automation. The articles were screened by two reviewers based on the inclusion criteria. Two reviewers were involved in the screening process at two stages namely, title-abstract and full text stages. In case of any discrepancies, it was sorted out through discussion or by contacting the third reviewer.

## Data Extraction

The following information was independently extracted from full texts by two reviewers (T.S and J.V) (i) the study characteristics(first author, country, the number of patients, age, body weight, dose, dosage regimen, sampling time, assay); (ii) the model characteristics and relevant covariates (study type, structural model, software, parameter estimation method, method of validation, criteria for addition/deletion of covariates and significant covariate(s); (iii) parameters of the model(fixed effect, random effect, their typical value and their Interindividual variability/Inter-occasion variability and residual variability).If there were any discrepancies, it was rectified by M.S or through discussion Tables 2 and 3.

## Quality assessment

 The reporting quality of the chosen PopPK studies of 5-FU was assessed using an adopted checklist derived from recently published guidelines for (i) population pharmacokinetic–pharmacodynamic studies [17], (ii) clinical pharmacokinetics [18]. As indicated in Table 4, the 36 criteria on the integrated updated checklist were divided into six categories: title, abstract, introduction/background, methods, results and discussion/conclusion. Each criteria received a score of 1 if the study’s pertinent data could be located; if not, it received a score of 0, if the criteria is not applicable to the study it was given NA. These standards were used to assess each of the selected PopPK investigations. The following formula was used to calculates each study’s compliance rate, and the outcome was shown as a percentage (Tables 4 and 5).

$$\:Compliance\:rate\:\left(\%\right)=\:\frac{Total\:no\:of\;\:criteria\:satisfied\:}{Total\:number\:of\:criteria}x\:100$$

**Table 2:** Characteristics of 5-FU population pharmacokinetic models and relevant covariates

No	Reference	Model	Software; parameter estimation method	Method validation	Tested covariates	Addition/deletion of covariates	Parameter (significant covariates)
5-FU IV							
1	Marie Sandstrom et al., (1996)	One-compartment (non-linear elimination)	NONMEM, version IV; FO	Goodness-of-fit plots	No tested covariates	NR	NR
2	M.-C. Etienne et al., (1998)	One-compartment (first order elimination)	NONMEM program, version IV, level 1.1	Linear regression	Age, sex, BSA, hepatic metastases, PMNC-DPD, liver enzymes, clock-time (8 a.m. versus 5 p.m.), elapsed time during CVI	*P* < 0.05; *P* < 0.001	Cl (age, low PMNC-DPD, high serum ALP and elapsed time during infusion)
3	FrancË oise Bressolle et al., (1999)	One-compartment	NONMEM computer program (Version 4.0, level 2.1); FO	Bayesian	Sex, BSA, age, BW, H, liver enzymes and serum creatinine	*P* < 0.05; *P* < 0.05	CL(Sex)
4	M. climente-marti et al., (2002)	One compartment (first order elimination)	NONMEM program (version V, level 1.0); FOCE	Bayesian	Age, gender, BW, IBW, H, BSA, CL_CR_, and hepatic function tests	*P* < 0.01; *P* < 0.01	Vd(IBW) and CL(BW)
5	M.A. Batey et al., (2002)	One compartment	NONMEM software (version V); FOCE	NR	Age, BW, creatinine, TB, AST, ALT, ALP	NR	CL(Age)
6	M. Sandstrom et al., (2006)	One- compartment model with saturable elimination	NONMEM version VI; FOCE-I	Goodness-of-fit plots	Age, BW, H, BSA, AST, ALT, ALB, TB, creatinine, CL_CR_	*P* < 0.05; *P* < 0.01	V(BSA); Vmax(CL_CR_)
7	Nagulu Malothu et al., (2010)	One-compartment (first order elimination)	NONMEM (version 5); FOCE-I	Goodness-of-fit plots	BW, H, age, dose and gender	NR	No influence of covariates
8	F. Mueller et al., (2013)	One-compartment (first order elimination)	NONMEM version VII; FOCE-I	Goodness-of-fit plots and Internal bootstrap test	Gender, age, BSA, CLCR, AST, ALT, albumin, TB and the DPYD genotype	*P* < 0.01; *P* < 0.01	CL(Gender)
9	Eduard Schmulenson et al., (2022)	One-compartment model with linear elimination	NONMEM^®^ version 7.2; FOCE-I	Goodness-of-fit plots, Internal bootstrap test and prediction- corrected visual predictive check	Age, sex, infusion time, CR, TB, ALT, AST, GGT, LDH, tumor markers (CA 19–9, CEA), and BSA	*P* < 0.05; *P* < 0.01	CL(BSA, Skeletal muscle index)
10	B.Porta-Oltra et al., (2004)	Two-compartment (first order elimination)	NONMEM package version V level 1.0; ELS	Goodness-of-fit plots and Internal bootstrap test	Age, BW, gender, IBW, LBM, BSA, Quetelet index, ALT, AST, GGT, ALP, LDH, TB, DB, ALT/ALP quotient, CR	*P* = 0.01; *P* = 0.005	No influence of covariates
11	Christian Woloch et al., (2012)	Two-compartment model	NONMEM version VI; FOCE-I	Visual Predictive Check	Age, sex, BW, H, CL_CR_ AST, ALT, GGT, histological status, BSA, LBM	*P* < 0.001	No influence of covariates
12	Usman Arshad et al., (2020)	Two-compartment with linear elimination	NONMEM 7.4.2; FOCE-I	Visual predictive checks	Age, BW, H, sex, BMI, LBW, BSA, ALT, AST and GGT, and albumin; and DPYD, TS and MTHFR genotypes.	*P* < 0.05; *P* < 0.05	CL(BSA)
13	Akihiro Yamada et al., (2025)	One -compartment model with zero-order input and first-order elimination	NONMEM^®^ (version 7.5.0)	Visual predictive checks	Zolbetuximab effect	NR; *P* = 0.001	No influence of covariates
**5-FU PRO-DRUGS**							
14	R. Gieschke et al., (2002)	One- compartment model (nonlinear elimination)	NONMEM (Version 5, level 1); FO	Goodness-of-fit plots	Age, BW, gender, race, BSA, CL_CR_, Cancer type, Liver metastasis, TB	*P* < 0.05; *P* < 0.001	CL(Cancer type)
15	Ronald Gieschke et al., (2003)	One-compartment	NONMEM (Version 5, level 1); FO	Goodness-of-fit plots	Age, BW, BSA, CL_CR_, gender, Race (Caucasian/black/other), Karnofsky scale (70/80/90–95/100), Liver metastasis, Albumin, AST, ALT, ALP, TB	*P* < 0.05	CL(ALP)
16	Emmanuelle Comets et al., (2003)	One-compartment	NONMEM version 5.1; FOCE-I	Goodness-of-fit plots and pseudo-residuals	BSA, sex, age, cancer type	*P* < 0.01; *P* < 0.01	Vd (BSA)
17	Saik Urien et al., (2005)	One-compartment	NONMEM (version V, level 1.1); FO	Goodness-of-fit plots and Internal bootstrap test	BW, BSA, H, age, TB, serum albumin	NR	No influence of covariates
18	Hyeong-Seok Lim et al., (2015)	One-compartment	NONMEM program (version IV, level 1.1); FOCE-I	Goodness of fit and Visual Predictive check	Plasma CDHP concentrations, sex, age, BW, CL_CR_ based on serum CR, and CL_CR_ based on 24-hour urine CR	*P* < 0.05	No influence of covariates
19	Esther Oyaga-Iriarte et al., (2019)	One-compartment	NONMEM	Goodness-of-fit plots and Visual Predictive Check	NR	NR	NR
20	Nastja Lunar et al., (2021)	One-compartment	Monolix^®^ (version 2019R1); SAEM	Visual Predictive Check	DPYD genotype, age, U, UH2/U ratio, CDA activity in plasma, BSA, ALP, GGT, AST, ALT, sodium concentration, kaliemia, CR, TB, albumin, plasma proteins and time between breakfast and capecitabine	*P* < 0.01; *P* < 0.001	K_el_ (UH2/U ratio)
21	S.Urien et al., (2003)	Two-compartmental model	Micropharm Population, MP2	Goodness-of-fit plots	Age, BW, BSA, ASAT, ALAT, ALP and TB.	*P* < 0.01; *P* < 0.01	No influence of covariates

*ALP* Alkaline phosphatase, *ALT* alanine aminotransferase, *AST* aspartate aminotransferase , *BMI* Body mass index, *BSA* Body surface area, *BW* Body weight, *CA* Carbohydrate antigen, *CDA* Cytidine deaminase, *CDHP *5-chloro-2,4-dihydroxypyridine, *CEA* Carcinoembryonic antigen, *Cl* apparent total body clearance of drug from central compartment, *CR* Creatinine, *CLCR* Creatinine clearance, *CVI* Continuous venous infusion, *DB* Direct bilirubin, *DPYD* Dihydropyrimidine dehydrogenase, *GGT* gamma-glutamyl transferase, *H* Height, *IBW* Ideal body weight, *LBM* Lean body mass, *LBW* Lean body weight, *LDH* Lactate dehydrogenase, *MTHFR* Methylenetetrahydrofolate reductase, *NR* Not reported, *PMNC-DPD* Peripheral mononuclear cell dihydropyrimidine dehydrogenase activity , *TB* Total bilirubin, *TS0* Thymidylate synthase, *Vd* apparent volume of distribution. *UH2* dihydrouracilemia, *U* uracilemia

**Table 3:** Fixed and random effect models of 5-FU population pharmacokinetic studies

No	References	Treatment protocol	Typical value (inter-individual variability, CV/%)					Residual variability	
			Type	Cl(L/h)		Vc(L)		Type	CV%/µg/ml
**5-FU** *i.v* **administration - One Compartment model**									
1	Marie Sandstrom et al., (1996)	epirubicin (EPI), fluorouracil (5 FU), and the cyclophosphamide	NR	79(30)		24(27)		NR	10
2	M.-C. Etienne et al.,(1998)	5-FU with cisplatin	Proportional	235(31)		NR		Proportional	19
3	FrancË oise Bressolle et al.,(1999)	5-FU with folinic acid	Additive	NR		21.2(105.8)		Proportional	41.6
4	M. climente-marti et al., (2002)	5-FU with oral levamisol	Exponential	92.44(71)		20.32(87)		Combined (proportional and additive)	1.89;0.234
5	M.A. Batey etl.al., (2002)	5-FU, methotrexate and cyclophosphamide	NR	26.3(10)		15.2		Combined (proportional and additive)	31; 0.00945
6	M. Sandstrom et al., (2006)	fluorouracil (5-FU)—epirubicin (EPI)—cyclophos phamide (CP)	NR	83.9(16.5)		23.2(9.6)		Proportional	11.5
7	Nagulu Malothu et al., (2010)	5-FU, methotrexate and cyclophosphamide	Additive	72.4(0.24)		12 (0.44)		Additive	6.76
8	F. Mueller et al., (2013)	5-FU	Proportional	158(21.9)		54.9(18.5)		Proportional	25.5
9	Eduard Schmulenson et al., (2022)	5-FU with folinate and oxaliplatin	Exponential	223(20.9)		46.1 (51.9)		Proportional	21.4
10	Akihiro Yamada et al., (2025)	5-FU	NR	179(34.4)		65.6(41.9%)		Proportional error	7.0
**5-FU** *i.v* **administration - Two Compartment model**									
**No**	**References**	**Treatment protocol**	**Typical value (inter-individual variability**,** CV/%)**					**Residual variability**	
			**Type**	**Cl(L/h)**	**Vc(L)**	**Vp(L)**	**Q(L/h)**	**Type**	**CV%/µg/ml**
11	B.Porta-Oltra et al., (2004)	5-FU with oral levamisol	Exponential	65.3(13.2)	14.7(11.8)	334(31.4)	19.6(25.5)	Additive	3
12	Christian Woloch et al., (2012)	5-FU plus leucovorin	Exponential	51(43)	22(50)	70	6(82)	Proportional	15
13	Usman Arshad et al., (2020)	5FU with cisplatin	NR	256(24.9)	5.85(130)	24	17.3	Proportional	0.36
**5-FU pro drug administration - One Compartment model**									
**No**	**References**	**Treatment protocol**	**Typical value (inter-individual variability**,** CV/%)**					**Residual variability**	
			**Type**	**CL(L/h)**		**Vc(L)**		**Type**	**CV%/µg/ml**
14	R. Gieschke et al., (2002)	Capecitabine	Proportional	1230(28)		17.8(FIX)		Combined (exponential and additive)	76; NR
			Proportional	1150(30)		17.8		Combined (exponential and additive)	81; NR
15	Ronald Gieschke et al., (2003)	Capecitabine	Proportional	1190(39.3)		17.8(fixed estimate)		Combined (exponential and additive)	58; NR
16	Emmanuelle Comets et al., (2003)	S1 (tegafur, potassium oxonate (oteracil) and gimeracil (CDHP))	Exponential	170(22)		9.4(22)		Combined	41
17	Saik Urien et al., (2005)	Capecitabine	NR	Capecitabine-218(18)		Capecitabine- 338(136)		Additive	0.64
18	Hyeong-Seok Lim et al., (2015)	S1 (tegafur, potassium oxonate (oteracil) and gimeracil (CDHP))	NR	26.80(22.5)		15.1(18.1)		Combined (proportional and additive)	0.0078,0.44
19	Esther Oyaga-Iriarte et al., (2019)	Capecitabine	Exponential	10.9		1 (FIX)		Combined (proportional and additive)	41.35;27.52
20	Nastja Lunar et al., (2021)	Capecitabine	NR	NO Cl		-		Combined (proportional and additive)	35;0.038
**5-FU pro drug administration - Two Compartment model**									
**No**	**References**	**Treatment protocol**	**Typical value (inter-individual variability**,** CV/%)**					**Residual variability**	
			**Type**	**Cl(L/h)**	**Vc(L)**	**Vp(L)**	**Q(L/h)**	**Type**	**CV%/µg/ml**
21	S.Urien et al., (2003)	UFT (tegafur and uracil)	Additive	5-FU and pro-drug 2.98(27.9)	5-FU and pro-drug 9.69(29.3)	5-FU and pro-drug 21.1(27.9)	5-FU and pro-drug 12.6(12.6)	Combined (proportional and additive)	11.5;2.5

*CL* apparent total body clearance of drug from central compartment, *CV* Coefficient of variation, *NR* Not reported, *Q* Inter-compartmental clearance between the central compartment and peripheral compartment, *Vc* apparent volume of central compartment, *Vp* apparent volume of peripheral compartment

### Data Analysis

The data analysis for forest plot was performed to visualize the covariate influence on PK parameters such as clearance (CL) and volume of central compartment (Vc) of 5-FU. The covariate effects were identified from the selected PopPK models for the systematic review. These covariates that has significance were transformed to fold change with lower and upper bounds to standardize these effects across the selected studies. The forest plot included both continuous and categorical covariates where power model and linear model was used respectively.

The fold change for the power model was computed as

$$Fold\;change\;relative\;to\;reference=\left(\frac{Covariate\;value}{Reference\;value}\right)^\beta$$

The lower and upper bounds were computed by replacing the estimate with lower and upper bounds of the confidence interval (CI). Similarly, the fold change for the linear model was calculated as,

$$\:Fold\;change\;relative\;to=1+\:\beta\:\:$$

Where β is the estimated continuous/ categorical covariate effect.

The 95% confidence interval for lower and upper bounds were calculated as (1 + lower CI) and (1 + upper CI) respectively. The dashed vertical line represents the reference value of 1 and the gray area (0.8–1.25) represents clinical significance [19]. The covariate’s point estimate and confidence interval that lie outside this range require consideration of dose adjustment. The entire data wrangling, fold change calculation, and the forest plot generation were conducted in R version 4.4.1.

## Results

**Fig. 1:**
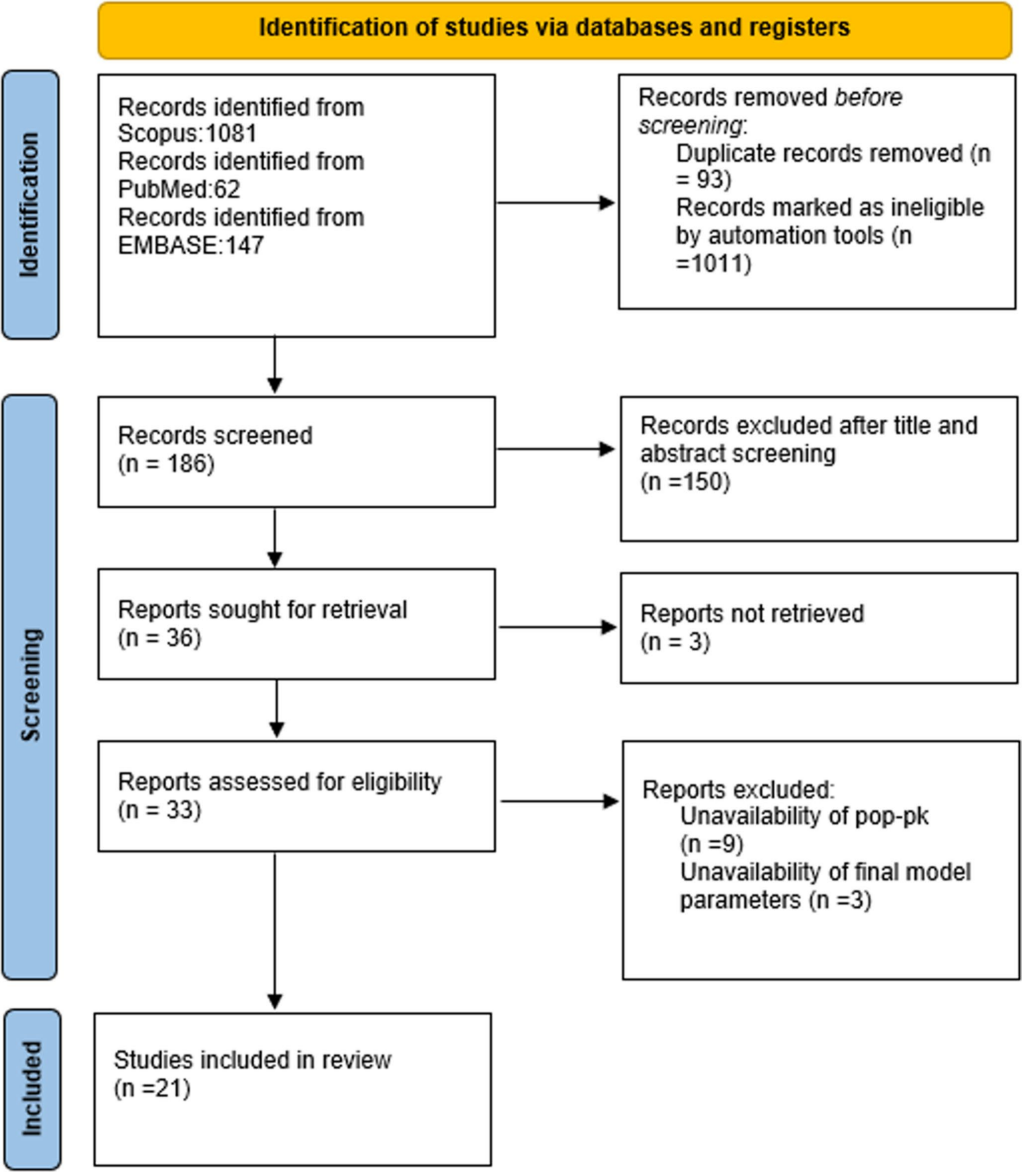
PRISMA flow chart representing the search results

Out of the 1290 articles identified, 62,1081 and 147 were from PubMed, Scopus and EMBASE respectively. A total of 93 duplicates were removed, and 1011 articles were identified to be ineligible and removed by automation tools. A total of 186 articles were screened for their title and abstracts, 150 were excluded. Out of the 36 articles, 3 articles weren’t retrieved leaving 33 articles. 12 were excluded for the following reasons (i) unavailability of PopPK models(*n* = 9); (ii)unavailability of final 5-FU model parameters(*n* = 3). Total 21 full text articles were identified for full text screening. This systematic review included 21 articles after full text screening. The screening process is depicted using a PRISMA flowchart in Fig 1.

## Characteristics of studies

13 articles are PopPK studies of 5-FU administered as *i.v* (bolus and infusion), 8 are PopPK studies of 5-FU administered as pro-drugs. All of these are indicated to breast cancer, colorectal cancer, head and neck, gastrointestinal and oesophageal cancer. The studies are conducted in different ethnicity including, Western, Asians and Caucasian population. The sample size of the studies ranges from 21 to 140 subjects for i.v administration and 7–78 subjects for pro-drugs in PopPK modelling.

The infusion time of 5-FU was different for different diseases, ranging from short *i.v* infusions (1–5 min) to 24 h. 5-FU administered as pro-drugs includes UFT, S-1 and Capecitabine. 5-FU in S-1 is typically dosed at 20–25 mg/kg or 300 mg/kg, while Capecitabine is administered at doses ranging from 825 to 2510 mg/m². During the sampling, blood was drawn at random/predetermined intervals. The 21 studies reported rigorous sampling schedules ranging greatly (e.g., every 15 to 30 min) to sparse designs (e.g., every several hours or days). Pre-dose, infusion completion, and many post-infusion intervals up to 12–24 h—some going up to 48–108 h—were typical time points. Depending on the regimen and study design, sampling was done on one or more days, usually on day 1 and other days (e.g., day 3, 4, 8, 15, or 21). Given the very short plasma half-life of 5-FU, (~ 10 to 15 min), sampling schemes requires dense early post dose measurements to provide information on the rapid distribution and elimination phases. The sampling schedule of within 2 h for *i.v* bolus; pre-dose and end of infusion for *i.v* infusion is ideal. 6 studies have reported intense sampling in the early distribution phase [14, 20–24] that characterises the early distribution and elimination phases and has captured covariate effects on these parameters. In contrast the late sampling, with measurements obtained at 9–24 h and extending to 36, 48, 73, or even 108 h after dosing, often during or long after continuous infusions, covariate detectability was likely constrained with either one or no covariates effects on the final model.

 5-FU concentrations are analysed by high pressure liquid chromatography–ultraviolet detection [11, 25], high pressure liquid chromatography [10, 14, 20–22, 26–31], liquid chromatography mass spectrometry tandem mass spectrometry [23, 24, 32, 33], reverse phase high pressure liquid chromatography–ultraviolet detection [34] and Gas Chromatography–Negative Ion Chemical Ionization–Mass Spectrometry [8].The characteristics/demographics of the 5-FU PopPK models are given in Table 1.

### Quality assessment

 As per the criteria, 4 studies from pro- drug and *i.v* administration collectively had a compliance rate of above 90%. The remaining 17 studies had a compliance rate of less than 90% (72.2 to 88.9%). Only 8 studies from the total of 21 studies reported external model validation. The criteria with lowest compliance rate include, Schematic of the final model (7.69%), Structural model (23.07%), Number of samples used for analyses (46.15%), Concentration vs. time plot (46.15%). Checklist for assessing the quality of 5-FU PopPK studies for *i.v* and pro-drugs are summarized in Tables 4 and 5 respectively.

**Table 4:** PRISMA checklist for assessing the quality of 5-FU i.v administration

Quality Criteria	Marie Sandstrom et.al.,1995	M.-C. Etienne et.al., 1998	FrancË oise Bressolle et.al., 1998	M. climente marti et.al., 2002	M.A. Batey et.al., 2001	B.Porta-Oltra et.al., 2004	M. Sandstrom et.al., 2005	Nagulu Malothu et.al., 2010	Christian Woloch et.al.,2012	F. Mueller et.al., 2013	Usman Arshad et.al.,2020	Eduard Schmulenson et.al., 2022	Akihiro Yamada et.al.(2025)	Compliance rate of each criterion (%)
					1									
Title														
The title identifies the drug(s) and patient population(s) studied	✓	x	✓	✓	x	✓	✓	✓	✓	✓	✓	x	✓	76.92
**Abstract**														
Name of the drug(s) studied	✓	✓	✓	✓	✓	✓	✓	✓	✓	✓	✓	✓	✓	100
Patient population studied	✓	✓	✓	✓	✓	✓	✓	✓	✓	✓	✓	✓	✓	100
Primary objective(s)	✓	✓	✓	✓	✓	✓	✓	✓	✓	✓	✓	✓	✓	100
Major findings	✓	✓	✓	✓	✓	✓	✓	✓	✓	✓	✓	✓	✓	100
**Background/introduction**														
Study rationale	✓	✓	✓	✓	✓	✓	✓	✓	✓	✓	✓	✓	✓	100
Specific objectives/hypothesis	✓	✓	✓	✓	✓	✓	✓	✓	✓	✓	✓	✓	✓	100
**Methods**														
Ethics approval	✓	x	x	✓	✓	✓	✓	✓	✓	✓	✓	✓	✓	84.61
Eligibility criteria of study participants	✓	✓	✓	x	✓	x	x	✓	x	✓	✓	✓	x	61.54
Co-administration or food	✓	✓	✓	✓	✓	✓	✓	✓	✓	✓	✓	✓	x	92.30
Dosing/frequency/formulation	✓	✓	✓	✓	✓	✓	✓	✓	✓	✓	✓	✓	x	92.30
Sampling time and frequency	✓	✓	✓	✓	✓	✓	✓	✓	✓	✓	✓	x	✓	92.30
Type of sample	✓	✓	✓	✓	✓	✓	✓	✓	✓	✓	✓	x	✓	92.30
Bioanalytical method	✓	✓	✓	✓	✓	✓	✓	✓	x	✓	✓	x	✓	84.61
Modeling software	✓	✓	✓	✓	✓	✓	✓	✓	✓	✓	✓	✓	✓	100
Modeling assumptions made	x	x	✓	x	✓	✓	✓	✓	✓	x	✓	✓	✓	69.23
Estimation method(s) used	✓	✓	✓	✓	✓	✓	✓	✓	✓	✓	✓	✓	✓	100
Structural model	x	x	x	x	✓	x	x	x	x	x	✓	x	✓	23.07
Co-variates tested	x	✓	✓	✓	✓	✓	✓	✓	✓	✓	✓	✓	✓	92.30
Co-variates analysis strategy	x	✓	x	✓	✓	✓	✓	✓	✓	✓	✓	✓	✓	84.61
Residual error model	✓	✓	✓	✓	✓	✓	✓	✓	✓	✓	✓	✓	✓	100
Methods for final model evaluation	x	✓	✓	✓	x	✓	✓	✓	✓	✓	✓	✓	✓	84.61
External model validation	NA	NA	✓	✓	NA	✓	✓	NA	NA	NA	✓	✓	NA	100
Model selection criteria (OFV, AIC, VPC etc)	✓	✓	✓	✓	✓	✓	✓	✓	✓	✓	✓	✓	✓	100
Number of study subjects	✓	✓	✓	✓	✓	✓	✓	✓	✓	✓	✓	✓	✓	100
Number of samples used for analyses	x	✓	x	x	x	x	✓	x	✓	x	✓	✓	✓	46.15
Equations for all model structures and covariates relationships	x	✓	✓	✓	✓	✓	✓	x	✓	✓	✓	✓	✓	84.61
**Results**														
Demographics details and clinical variables	✓	✓	✓	✓	✓	✓	✓	✓	✓	✓	✓	✓	✓	100
Concentration vs. time plot	x	x	✓	x	✓	✓	✓	x	x	x	✓	✓	x	46.15
Schematic of the final model	x	x	x	x	x	x	x	x	x	x	✓	x	x	7.69
Table of final model parameters	✓	x	✓	✓	✓	✓	✓	✓	✓	✓	✓	✓	✓	92.30
Summary of the model building process and the derived final model	✓	✓	✓	✓	✓	✓	✓	✓	✓	✓	✓	✓	✓	100
Final model evaluation plots	✓	x	✓	✓	x	✓	✓	✓	✓	✓	✓	✓	✓	84.61
A description of simulation results or scenarios(if applicable)	x	x	✓	✓	x	✓	✓	✓	✓	✓	✓	✓	✓	76.92
**Discussion/conclusion**														
Study limitations	✓	✓	✓	✓	✓	✓	x	x	x	✓	x	✓	✓	69.23
Study findings	✓	✓	✓	✓	✓	✓	✓	✓	✓	✓	✓	✓	✓	100
**Total compliance rate of each study(%)**	72.22	74.2	86.11	83.33	82.85	88.89	88.89	82.85	82.85	85.71	97.22	83.33	85.71	

√ denotes study reported the quality criteria, × denotes study did not report the quality criteria, and NA Not applicable

**Table 5:** PRISMA checklist for assessing the quality of 5-FU i.v administration

Quality Criteria	*R*. Gieschke et al., 2002	Ronald Gieschke et al., 2003	S.Urien et al., 2003	Emmanuelle Comets et al., 2003	Saik Urien et al.,2005	Hyeong-Seok Lim et al.,2015	Esther Oyaga-Iriarte et al., 2019	Nastja Lunar et al., 2021	Compliance rate of each criterion(%)
Title									
The title identifies the drug(s) and patient population(s) studied	x	✓	✓	✓	✓	✓	x	✓	75
**Abstract**									
Name of the drug(s) studied	✓	✓	✓	✓	✓	✓	✓	✓	100
Patient population studied	✓	✓	✓	✓	✓	✓	✓	✓	100
Primary objective(s)	✓	✓	✓	✓	✓	✓	✓	✓	100
Major findings	✓	✓	✓	✓	✓	✓	✓	✓	100
**Background/introduction**									
Study rationale	✓	✓	✓	✓	x	✓	✓	✓	87.5
Specific objectives/hypothesis	✓	✓	✓	✓	✓	✓	✓	✓	100
**Methods**									
Ethics approval	x	✓	✓	✓	✓	✓	x	✓	75
Eligibility criteria of study participants	x	✓	✓	✓	x	✓	x	x	50
Co-administration or food	✓	✓	✓	✓	✓	x	x	x	62.5
Dosing/frequency/formulation	✓	✓	✓	✓	✓	✓	✓	✓	100
Sampling time and frequency	✓	✓	✓	✓	✓	✓	x	✓	87.5
Type of sample	✓	✓	✓	✓	✓	✓	✓	✓	100
Bioanalytical method	✓	✓	✓	✓	✓	✓	✓	✓	100
Modeling software	✓	✓	✓	✓	✓	✓	✓	✓	100
Modeling assumptions made	✓	✓	✓	✓	✓	✓	✓	✓	100
Estimation method(s) used	✓	✓	x	✓	✓	✓	x	✓	75
Structural model	✓	✓	✓	✓	✓	x	✓	✓	87.5
Covariates tested	✓	✓	✓	✓	✓	✓	x	✓	87.5
Covariate analysis strategy	✓	✓	✓	✓	✓	✓	x	✓	87.5
Residual error model	✓	✓	✓	✓	✓	✓	✓	✓	100
Methods for final model evaluation	✓	✓	✓	✓	✓	✓	✓	✓	100
External model validation	NA	✓	NA	✓	NA	NA	NA	NA	100
Model selection criteria (OFV/AIC, etc.)	✓	✓	✓	✓	✓	✓	✓	✓	100
Number of study subjects	✓	✓	✓	✓	✓	✓	✓	✓	100
Number of samples used for analyses	x	✓	✓	✓	✓	✓	✓	✓	87.5
Equations for all model structures and covariates relationships	✓	✓	x	✓	✓	✓	x	✓	75
**Results**									
Demographics details and clinical variables	✓	✓	✓	✓	✓	✓	✓	✓	100
Concentration vs. time plot	✓	x	✓	✓	✓	✓	✓	x	75
Schematic of the final model	✓	✓	✓	✓	✓	x	✓	✓	87.5
Table of final model parameters	✓	✓	✓	✓	✓	✓	✓	✓	100
Summary of the model building process and the derived final model	✓	✓	✓	✓	✓	✓	✓	✓	100
Final model evaluation plots	✓	✓	✓	✓	✓	✓	✓	✓	100
A description of simulation results or scenarios(if applicable)	x	x	x	✓	x	✓	✓	x	37.5
**Discussion/conclusion**									
Study limitations	✓	✓	x	✓	x	✓	✓	✓	75
Study findings	✓	✓	✓	✓	✓	✓	✓	✓	100
**Total compliance rate of each study(%)**	86.11	94.44	88.89	100	88.89	91.67	75	88.89	

√ denotes study reported the quality criteria, × denotes study did not report the quality criteria, and NA Not applicable

### Characteristics of PopPK models

Nineteen studies developed PopPK models using NONMEM software with first-order conditional estimation method (FOCE), first-order conditional estimation method with interaction (FOCE-I), first order estimation method (FO), Extended Least Squares regression (ELS), Stochastic Approximation Expectation Maximization (SAEM). Additionally, other software such as Monolix, Micropharm Population, MP2 were also utilized [11, 31]. 9 studies from 5-FU administered as *i.v* developed a one compartment structural model [20–27, 29], 3 studies developed a two compartment model [14, 34, 35]. Out of the eight studies for pro-drugs, one study [11] has used two-compartment model, 7 studies reported one-compartment structural model. The metabolite structural model, in which Tegafur, Capecitabine, UFT and S-1 undergo metabolic activation to generate 5-FU, was commonly used in the pharmacokinetic modelling of 5-FU pro-drugs in the included research. Multiple compartments were included in these models to depict the pro-drugs and their active metabolite 5-FU’s sequential metabolism and elimination. In contrast, 5-FU itself was most often characterized using a one-compartment model, reflecting its rapid distribution and elimination kinetics. Across the 21 studies, 8 studies did not report the type of model used for IIV. 6 studies reported residual variability as exponential error model, 5 studies reported proportional error model and additive error model was reported the least for 2 studies. 2 studies have reported non- linear pharmacokinetics of 5-FU [11, 27].

The residual variability was explained by proportional (coefficient of variation%, 7.0–41.6.0.6%), additive error model (0.64–6.76 mcg/ml), combination (exponential and additive; 58–81%), and combined (proportional and additive; 0.5–68.87.5.87%). For 5-FU *i.v.* models, typical value and IIV of CL ranged from 8.847 to 256 L/hr and 0.24–71.24% respectively. The Vc was obtained at a range of 5.85–54.9 L with the IIV of 0.44–130%. 3 studies from the 5-FU *i.v* administration was developed using a two compartmental model with Vc, Vp and Intercompartmental clearance (Q) ranging from 24 to 334 L and 6–19.6 L respectively. Study including UFT had a CL, Vc, Vp and Q, 2.98,9.69,21.2,12.6 respectively. Only 2 studies from pro-drugs reported elimination rate constant, hence this review didn’t include the parameter. The typical value and IIV of CL for pro-drug administration of 5-FU ranged from 10.9 to 1230 L/hr and 18–39.3.3% for capecitabine, 26.8–170 L/hr and 22% for S-1. The Characteristics and Parameters of PopPK models are summarized in Tables 2 and 3.

### Influence of covariates on PK parameters of 5-FU

The covariate selection was stepwise analysis with eight papers reporting forward addition (*p* < 0.05) with an objective function value ≥ 3.84 [10, 22, 25, 26, 32–34, 36] and six studies have reported backward elimination (*P* < 0.01) with a objective function value ≥ 10.83 [8, 20, 22, 23, 36, 37]. Five studies did not report the covariate selection analysis [11, 21, 27, 30, 38].

The following covariates affect CL of 5-FU: age [21, 22], weight [20], gender [25], alkaline phosphatase(ALP) [26, 33], infusion time [22], BSA [34, 39], SMI [39], peripheral mononuclear cell dihydropyrimidine dehydrogenase (PBMC-DPD) activity [26], cancer type [32], Ideal body weight [20]. The other covariates that were tested for both *i.v* and pro-drug administration includes creatinine clearance, liver enzymes(alanine transaminase (ALT), aspartate transaminase (AST), g-glutamyl transpeptidase (gGT), ALP, lactate dehydrogenase (LDH), total and direct bilirubin (Bt, Bd)), dose, ALT/ALP quotient, creatinine, Race (Caucasian/black/other), Karnofsky scale (70/80/90–95/100), albumin, plasma proteins (all assessed at baseline). Eight models did not report any covariate relationship with PK parameters [10, 11, 14, 22, 28, 30, 35, 38].

The significance of covariate/covariate effect on PK parameters- CL and Vc were summarized in a forest plot. Fold changes and confidence intervals were calculated from reported estimates and displayed stratified by study and covariate type.

Of the 21 included studies, only 8 reported covariate effect estimates suitable for forest plot construction. Among these, 2 studies examined categorical covariates, specifically cancer type [32] and gender [25], on CL. 1 study evaluated the effect of body surface are BSA [27] on Vc. Continuous covariates influencing CL included weight [20], BSA [34, 39], SMI [39], and alkaline phosphatase (ALP) [33]. Among the 2 studies that has reported non- linear pharmacokinetics of 5-FU one study [11] reported no significance on PK parameters and Sandstrom et al., reported significance of creatinine clearance on Vmax [27].

### Forest Plot Analysis

Forest plot was constructed by using the data from the studies that were included for the systematic review. Out of the 8 studies that were utilized for the construction of forest plot, most of the covariates which includes cancer type, gender, ALP, SMI, BSA, weight showed significant effects on CL and one study incorporated BSA on Vc. Bressolle et al. (1999) reported effect of males having nearly a 3-fold higher CL compared with females [25]. This represents the largest effect size observed and suggests potential sex-based differences in 5-FU metabolism, possibly mediated by dihydropyrimidine dehydrogenase (DPD) activity variations. Body weight also showed clinical relevance, with maximum weight (110.5 kg) associated with a 56% increase in CL (95% CI: 1.37–1.79), while minimum weight (50 kg) resulted in a 40% reduction, indicating substantial exposure variability across weight extremes. Gieschke et al.(2002) showed variable effects with higher ALP was associated with 19% decreased CL (95% CI: 0.93 − 0.70), while minimum ALP (44 U/l) increased CL by 22% (95% CI: 1.07–1.39) [32]. In contrast, Maximum BSA (2.85 m²) and maximum SMI (8.41 cm²/m²) were associated with increased CL by 34% (95% CI: 1.23–1.46) and 5% (95% CI: 1.02–1.07) respectively. This suggests that BSA is a sensitive predictor than SMI alone. BSA also influenced the volume of distribution. Maximum BSA (2.1 m²) was associated with 23% (95% CI: 1.14–1.32) higher Vc while minimum BSA (1.3 m²) decreased Vc by about 35% (95% CI: 0.76 − 0.56). This suggests that there is an effect of BSA in both elimination and distribution processes of 5-FU. The covariate effects, body weight and sex had the 95% CI outside the range (80–125%) signifying clinical relevance on CL of 5-FU.

**Fig. 2:**
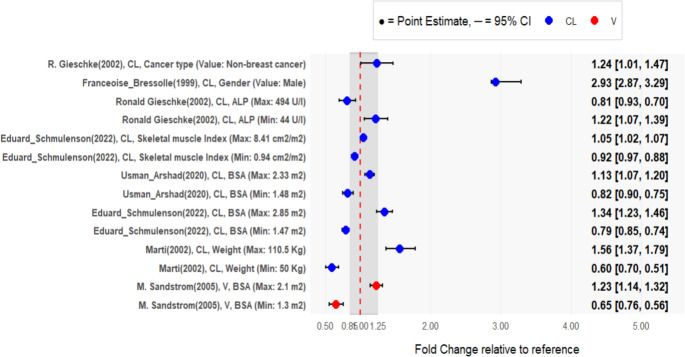
Forest plot representing the covariate effect on PK parameters of 5-FU

Cancer type has showed a 24% higher CL (95% CI: 1.01–1.47) in non-breast cancer patients compared to the breast cancer patients. The CI barely excludes the reference range suggesting limited clinical impact. The width of the 95% confidence intervals varied considerably across the studies due to differences in the sample sizes and inter individual variability. The wider CI for sex and weight despite clinical significance suggest high variability in the weight – clearance relationships at extremes (1.37–1.79), possibly due to altered body composition (increased adipose vs. lean mass) that simple weight metrics fail to capture. The constructed forest plot is given in Fig. 2.

## Discussion

To the best of our knowledge this is the first systematic review that summarizes the PopPK of 5-FU in adult population. This review provides a comprehensive summary of published PopPK models of 5-FU for i.v and pro-drug administrations (capecitabine, UFT, and S-1).

Across the 21 included models, body size metrics were the most consistently identified covariates on CL: BSA was found to be significant in 3 out of 4 studies, body weight in 2 out of 3, and ALP in 2 out of 3. These covariates showed consistent direction of effect across independent study populations (positive for BSA and weight, inverse for ALP). Sex was found to be significant in only 1 out of 2 studies that tested it, and SMI in the single study that assessed it. Age was reported as significant covariate in two studies, with increasing age associated with reduced CL consistent with age-related decline in hepatic blood flow and liver mass given that 5-FU is a highly extracted drug (93%) [21, 26]. However, its clinical significance could not be analysed using forest plot analysis due to insufficient data across studies. Sex, body weight, and SMI are correlated covariates, with males consistently exhibiting higher CL alongside greater SMI and body weight. This alignment suggests that these metrics capture overlapping aspects of body composition and metabolic capacity rather than independent pharmacokinetic determinants. The consistency of direction for body size metrics and ALP across independent populations strengthens confidence in these as genuine pharmacokinetic determinants. However, a striking sex effect reported by Bressolle et al. (1999, fold change 2.93) stands as an outlier that no other study replicates and requires confirmation before incorporation into dosing algorithms.

As quantified in the forest plot (Fig. 2), body weight and sex produced covariate effects with 95% CIs falling entirely outside the 80–125% range, confirming clinical relevance [19]. BSA produced moderate effects, while ALP effects were clinically modest but consistent. SMI produced the smallest effect, suggesting BSA is a more sensitive body composition predictor than SMI alone. For Vc, two additional studies reported covariate effects including BSA [27] and ideal body weight [20]. However, these effects were attributed to a reduction in IIV rather than significantly impacting the parameter itself. Both BSA and ideal body weight are considered poor predictors of Vc for 5-FU, given its hydrophilic nature, which means that Vc correlates more closely with lean body mass and total body water than with conventional body size metrics [40]. Gusella and colleagues confirmed this, reporting that total body water and fat-free mass were statistically superior predictors of Vc compared to BSA and body weight [41].

The covariate effects observed in the forest plot reflect not only genuine pharmacokinetic relationships, but also the methodological fingerprints of each contributing study’s sampling design. A distinction that becomes especially consequential given 5-FU’s ultra-short plasma half-life of approximately 8–20 min, where even minor deviations in sampling timing relative to end of infusion can disproportionately distort CL estimates [42].

The forest plot is dominated by CL-covariates (12 out of 14 entries) with only Sandstrom et al. (2006) reporting a significant covariate on Vc. This asymmetry is directly attributable to sampling design. The studies that captured exclusively steady-state concentrations during prolonged infusions, such as Arshad et al. (2020) with only four samples during a 5-day infusion, yielded implausibly high IIV on Vc (130% CV with a typical value of 5.85 L) indicative of non-identifiability rather than true variability. In contrast, Sandstrom et al. (2006), who sampled at 0.17, 1, 6, and 15–24 h post-dose capturing the rapid elimination phase, achieved the lowest IIV on Vc (9.6% CV) and sufficient precision to detect BSA on Vc. Due to sub-optimal sampling design, the estimate of Vc and the impact of covariates can be inadequately explained. Therefore, it was noted that there were few studies that reported the impact of covariates on Vc. The heterogeneity in CL covariate effect sizes is similarly shaped by sampling informativeness. Studies relying on sparse steady-state sampling reported higher unexplained IIV on CL (Arshad et al. 24.9%; Yamada et al. 34.4%) compared with designs capturing both infusion and post-infusion phases (Sandstrom et al. 16.5%) [27]. These results suggests that sampling-driven differences in individual parameter precision systematically distort apparent covariate effect sizes across studies.

The absence of DPD/DPYD from the PopPK covariate synthesis warrants particular attention given that DPYD genotyping has become an established pre-treatment screening tool in clinical practice [43] Current Clinical Pharmacogenetics Implementation Consortium (CPIC) and Dutch Pharmacogenetics Working Group (DPWG) guidelines recommend dose reductions of 25–50% for carriers of clinically actionable DPYD variants, including DPYD* 2 A (c.1905 + 1G > A)*,* c.1679T > G (DPYD*13), c.2846 A > T, and the HapB3 haplotype (with c.1129–5923 C > G identified as the causative variant), with pre-treatment DPYD testing now mandated in several European countries [44]. The disconnect between this established clinical importance and the failure of DPD to emerge as a significant covariate in the identified PopPK models can be attributed to several factors. Most of the included studies were conducted prior to the widespread adoption of DPYD testing and genotyping. Assays such as PBMC-DPD activity measurement and the uracil/dihydrouracil (UH2/U) ratio were neither standardised across centres nor routinely incorporated into pharmacokinetic study protocols. Beyond data availability, the statistical capacity to detect DPD effects was fundamentally constrained by low variant prevalence (2–3%, yielding fewer than 3–4 expected carriers in study sample sizes of 24–140 patients), and by sampling designs not optimised for 5-FU’s ultra-short half-life that compress true DPD-driven inter-individual differences through high empirical Bayes shrinkage [45].

Out of the four studies that evaluated the effect of DPD on 5-FU PK [23, 26, 31, 34], only one reported DPD as significant on 5-FU CL [26], a finding consistent with these power and design limitations rather than an indication that DPD lacks pharmacokinetic relevance. Importantly, the correlated nature of covariates identified in this review raises the possibility that DPD variation may be partly captured by other covariates that did reach significance. The approximately 15% lower DPYD activity reported in women compared with men suggests that the large sex effect on CL observed by Bressolle et al. (1999) may partly reflect unmeasured differences in DPD activity rather than an independent sex effect. Similarly, body composition metrics such as body weight and SMI, which correlate with sex and showed significance on CL, could serve as indirect surrogates for DPD-driven variability, given that males typically have higher lean mass, higher DPD activity, and correspondingly higher CL [40]. This implies that the covariate effects quantified in the present review may partly capture underlying DPD variation rather than representing independent pharmacokinetic determinants. However, this hypothesis cannot be tested with the available data, as no included study simultaneously evaluated DPD alongside sex or body composition covariates in the same model. DPD, therefore remains absent from the covariate synthesis not because it lacks biological importance but because these studies were neither designed nor powered to detect it. This represents the most consequential evidence gap identified in this review and reinforces the need for future PopPK studies that prospectively integrate DPYD genotyping or phenotypic DPD assessment alongside anthropometric and biochemical covariates within adequately powered study designs.

Quantifying the proportion of inter-individual variability in 5-FU CL explained by covariates versus residual unexplained variability is essential for determining whether covariate-based dosing can substitute for therapeutic drug monitoring [46]. Across the included intravenous 5-FU studies, IIV on CL in the final covariate models ranged from 10% to 71% CV, with majority of studies reporting values between 20% and 35% CV (Etienne et al. 1998, 31%; Arshad et al. 2020, 24.9%; Schmulenson et al. 2022, 20.9%; Mueller et al. 2013, 21.9%; Yamada et al. 2025, 34.4%). For pro-drug administrations, residual IIV on CL ranged from 18% to 39.3% CV for capecitabine and 22% CV for S-1, indicating that the metabolic conversion of pro-drugs to active 5-FU introduces an additional source of variability that covariate adjustment similarly fails to resolve. Although most included studies did not report IIV values before addition of covariates, preventing direct calculation of the proportion of variability explained. The target AUC-guided dosing aims for 20–24 mg·h/L and exposures outside this range are associated with significantly increased toxicity and reduced response [47]. A residual IIV of 20–35% CV on CL, even after covariate adjustment, translates into a range of individual exposures spanning from subtherapeutic to potentially toxic levels under a fixed covariate-adjusted dose. Even in the best-case scenario (Batey et al. 2002, residual IIV 10% CV), this was achieved in a study that found no significant covariates, suggesting that the low IIV reflects a homogeneous population or dense sampling rather than successful covariate explanation. This indicates that BSA-based dosing, even supplemented with covariate adjustments, cannot achieve the precision required for 5-FU’s narrow therapeutic index, and supports the need for therapeutic drug monitoring for majority of patients receiving 5-FU.

The dose and length of infusion for i.v. administered 5-FU also significantly influences the nonlinear relationship between plasma concentration and pharmacokinetics [48]. Clinical toxicity was significantly lower with 1-hour infusion compared to the same dose delivered over 5-minute duration [49]. Jean et al. reported lower CL and higher Cmax and AUC with 5-FU administered over 5 min compared to a one-hour infusion at 370 mg/m², attributable to immediate peaks after drug administration and saturated DPD enzyme activity, necessitating longer infusions to avoid adverse impact on anti-tumour activity. Hepatic metabolism accounts for approximately 80% of 5-FU elimination, with only 3% of the dose reaching the tumour and the majority catabolised into inactive metabolites by DPD in the liver [50]. Pro-drugs are activated by hepatic enzymes and occasionally by tumour tissues, and liver impairment resulting in elevated ALP levels has been reported to reduce CL, with elevated disease progression associated with high 5-FU exposure [26].

The clinical relevance of the covariate effects identified in this review can be contextualised against established exposure-response relationships for 5-FU. Lee et al. (2016) reported that under standard BSA-based dosing, only 21% of patients achieve the target AUC of 20–24 mg·h/L, with 51% falling below and 28% exceeding this range [42]. Since AUC is inversely proportional to CL at a fixed dose, covariates that substantially alter CL will shift individual patients further from the therapeutic window.

The forest plot estimates (Fig. 2) demonstrate that several covariates impact CL. Body weight extremes yielded fold changes of 0.60 to 1.56 on CL (Climente-Marti et al. 2002), indicating that a low-weight patient (50 kg) would have approximately 40% lower CL and correspondingly higher exposure than a patient at the reference weight. Conversely, a high-weight patient (110.5 kg) would have 56% higher CL with proportionally lower exposure. The sex effect reported by Bressolle et al. (1999) showed a 2.93-fold difference in CL between males and females. Elevated ALP was associated with a 19% reduction in CL (fold change 0.81; Gieschke et al. 2002). Given that BSA-based dosing already fails to achieve target exposure in majority of patients, these covariate-driven shifts would further widen the gap between achieved and target AUC [42, 50].

These findings suggest a role for covariate-based risk stratification in clinical settings where universal TDM is not feasible. Patients with multiple characteristics that reduce the CL particularly low body weight, female sex, and elevated ALP should be prioritised for early-cycle pharmacokinetic monitoring to detect and correct toxic overexposure. Conversely, high-weight male patients with large BSA may require early TDM to identify subtherapeutic exposure and guide dose escalation. Patients within the central range of these covariates may be managed initially with standard BSA-based dosing, with TDM reserved for those experiencing unexpected toxicity or inadequate response.

Although BSA-based dosing remains the standard strategy, a comparative covariate analysis reported that indices representing body mass showed no significant superiority over BSA, and studies increasingly recommend accounting for fat-free mass, duration of infusion, and hepatic function markers alongside BSA. Taken together, the findings of this review indicate that inter-individual variability in 5-FU exposure is more strongly associated with biological and physiological factors than body surface area alone. The covariates identified in this review are insufficient to replace therapeutic drug monitoring, but they can inform initial dose selection, identify patients at highest risk of extreme exposure, and guide the prioritisation of pharmacokinetic monitoring to optimise safety and efficacy.

### Future Directions

This review consolidates the PK parameters and the effect of covariates that serves as a unified reference for model comparison and provides a framework for development of predictive performance of PopPK models of 5-FU. These model frameworks support future model-informed precision dosing strategies for 5-FU-based therapies.

### Limitations

The review papers included studies only in English and excluded other languages. Most of the studies included summarizes the PopPK models and doesn’t discuss the generalizability which can be useful for model informed dosing decisions in clinical practice. Besides, most of studies used clinical data from other studies, hence it could result in bias in the data used for modelling. The pharmacogenetics of 5-FU was either not addressed or reported insignificant on the PK parameters in the included studies restricting the potential of the systematic review. Only 40% of the studies have been included in the forest plot analysis due to insufficient information regarding the covariates required for analysis. The papers didn’t report few characteristics leading to missing information from the PopPK models. The inconsistent availability of DPYD genotype or DPD activity data limited the ability to assess the contribution of this pathway to variability in 5-FU exposure across studies.

## Conclusion

This systematic review of 21 PopPK models demonstrates that residual variability on clearance remains between 20% and 35% CV in majority of studies even after covariate incorporation, providing quantitative evidence that covariate-based dose adjustment alone cannot achieve the precision required within the therapeutic window of 20–24 mg·h/L. Among the covariates identified, body weight and sex produced effects of unequivocal clinical magnitude with clearance varying up to 3-fold by sex and 56% across weight extremes, while ALP showed consistent inverse effects. These covariates can inform initial dose selection and serve as triage criteria for early-cycle TDM, with low-weight females with elevated ALP at highest risk of toxic overexposure and high-weight males at greatest risk of subtherapeutic exposure. However, evidence regarding IIV prior to addition of covariates was not reported in most studies, DPD could not be evaluated due to insufficient genotypic data and sampling designs were not optimised for shorter half-life of 5-FU. Future PopPK studies should incorporate dense early post-infusion sampling to improve the identifiability of distribution and elimination parameters. These studies should also integrate DPYD genotyping, with deliberate enrichment for variant carriers to ensure adequate statistical power and include external validation as a minimum methodological standard. Until such evidence is generated, the findings of this review support a three-tier dosing framework where BSA-based dosing provides initial dose calculation. Covariate profiles, particularly body weight, sex, and ALP must guide risk stratification to identify patients at highest risk of extreme exposure and therapeutic drug monitoring within the first treatment cycle achieves and maintains target exposure within the therapeutic window of 20–24 mg·h/L.

## Supplementary Information

Below is the link to the electronic supplementary material.

Supplementary Material 1 (DOCX 15.5 KB)

## Data Availability

The current study is based on the published literature and is already available as open access.

## Abbreviations

5-FU: 5-Fluorouracil
ALP: Alkaline phosphatase
ALT: Alanine transaminase
AST: Aspartate transaminase
Bd: Direct bilirubin
BSA: Body Surface Area
Bt: Total bilirubin
CDHP: 5-chloro-2,4-dihydroxypyridine
CI: Confidence interval
CL: Clearance
CVI: Continuous venous infusion
DPD: Dihydropyrimidine dehydrogenase
DPYD: Dihydropyrimidine dehydrogenase gene
FO: First order estimation method
FOCE-I: First order conditional estimation method with interaction
gGT: g-glutamyl transpeptidase
GoF: Goodness of fit
IIV: Interindividual variability
LDH: Lactate dehydrogenase
PBMC-DPD: Peripheral mononuclear cell dihydropyrimidine dehydrogenase
PK: Pharmacokinetics
PopPK: Population pharmacokinetics
PRISMA: The Preferred Reporting Items for Systematic Reviews and Meta analyses
Q: Intercompartmental clearance
S-1: Tegafur/gimeracil/oteracil
SMI: Skeletal muscle index
TDM: Therapeutic drug monitoring
UFT: Tegafur/uracil
Vc: Volume of central compartment,
Vd: Volume of distribution
Vp: Volume of peripheral compartment
VPC: Visual predictive check
