# Supplementary material for: Factors influencing pharmacokinetics of 5-fluorouracil in cancer patients: a systematic review of population pharmacokinetic models
Source: Eur J Clin Pharmacol. 2026 Apr 15;82(5):126. doi: 10.1007/s00228-026-04043-5 (PMC13083512; doi:10.1007/s00228-026-04043-5)
Supplement: Supplementary file 1 — Supplementary Material 1 (DOCX 15.5 KB) [file 228_2026_4043_MOESM1_ESM.docx]

**FACTORS INFLUENCING PHARMACOKINETICS OF 5-FLUOROURACIL IN CANCER PATIENTS: A SYSTEMATIC REVIEW OF POPULATION PHARMACOKINETIC MODELS**

**JOURNAL NAME - CANCER CHEMOTHERAPY AND PHARMACOLOGY**

Teshini Suthahar^1^, Jayashree Veerabhadrappa^2^, Renuka Munshi^3^, Elstin Anbu Raj S^1,4^, Vikram Gota^5^, Vijay Ivaturi^6^, Surulivelrajan Mallayasamy^*^

^1,2^Department of Pharmacy Practice, Manipal College of Pharmaceutical Sciences, Manipal Academy of Higher Education, Manipal 576 104, Karnataka, India.

^3^Department of Clinical Pharmacology, TN Medical College & BYL Nair Hospital, Dr. AL Nair Road, Mumbai Central, Mumbai 400 008

^4^ Centre for Evidence-informed Decision-making, Prasanna School of Public Health, Manipal Academy of Higher Education, Manipal 576 104, Karnataka, India.

^5^Department of Clinical Pharmacology, Advanced Centre for Treatment, Research and Education in Cancer, Mumbai, India.

^6^Centre for Pharmacometrics, Department of Pharmacy Practice, Manipal College of Pharmaceutical Sciences, Manipal Academy of Higher Education, Manipal 576 104, Karnataka, India.

*Corresponding Author - Surulivelrajan Mallayasamy,

Email: [msv.rajan@manipal.edu](mailto:msv.rajan@manipal.edu)

Address: Department of Pharmacy Practice, Manipal College of Pharmaceutical Sciences, Manipal Academy of Higher Education, Manipal 576 104, Karnataka, India

**Table I Search strategy**

| **Database** | **Search Strategy** | **No of articles** |
| --- | --- | --- |
| PubMed | ((("neoplasm*"[MeSH Terms]) OR (cancer[Text Word])) AND (("fluorouracil"[MeSH Terms]) OR ("5-fluorouracil"[Text Word]))) AND ((("population pharmacokinetic*") OR ("pop pk")) OR ("NONMEM")) | **58** |
| Scopus | ( INDEXTERMS ( neoplasms ) OR TITLE-ABS-KEY ( cancer ) ) AND ( INDEXTERMS ( fluorouracil ) OR TITLE-ABS-KEY ( "5 fluorouracil" ) ) AND ( INDEXTERMS ( pharmacokinetics ) OR INDEXTERMS ( pharmacokinetics ) OR TITLE-ABS-KEY ( pharmacokinetics ) ) AND ( ALL ( "pop pk" ) OR ALL ( "population pharmacokinetics" ) ) | **1030** |
| Embase | (((neoplasm*/exp) OR (cancer)) AND ((fluorouracil/exp) OR (5-fluorouracil))) AND ((('population pharmacokinetic*' ) OR ('pop pk' )) OR (NONMEM )) | **131** |
